# New perspectives of nitric oxide donors in cardiac arrest and cardiopulmonary resuscitation treatment

**DOI:** 10.1007/s10741-013-9397-4

**Published:** 2013-05-28

**Authors:** Peter Kruzliak, Olga Pechanova, Tomas Kara

**Affiliations:** 1 Institute of Normal and Pathological Physiology and Centre of Excellence for Regulatory Role of Nitric Oxide in Civilization Diseases, Slovak Academy of Sciences, Bratislava, Slovak Republic; 2 Department of Cardiovascular Diseases, International Clinical Research Center, St. Anne′s University Hospital and Masaryk University, Pekarska 53, 656 91 Brno, Czech Republic; 3 Division of Cardiovascular Diseases, Mayo Clinic and Mayo College of Medicine, Rochester, MN USA

**Keywords:** Nitric oxide, Nitric oxide donors, Adenosine, Cardiac arrest, Cardiopulmonary, Resuscitation

## Abstract

Nitric oxide (NO) is often used to treat heart failure accompanied with pulmonary edema. According to present knowledge, however, NO donors are contraindicated when systolic blood pressure is less than 90 mmHg. Based on recent findings and our own clinical experience, we formulated a hypothesis about the new breakthrough complex lifesaving effects of NO donors in patients with cardiac arrest and cardiopulmonary resuscitation therapy. It includes a direct hemodynamic effect of NO donors mediated through vasodilation of coronary arteries in cooperation with improvement of cardiac function and cardiac output through reversible inhibition of mitochondrial complex I and mitochondrial NO synthase, followed by reduction in reactive oxygen species and correction of myocardial stunning. Simultaneously, an increase in vascular sensitivity to sympathetic stimulation could lead to an increase in diastolic blood pressure. Confirmation of this hypothesis in clinical practice would mean a milestone in the treatment for cardiac arrest and cardiopulmonary resuscitation.

## Introduction

Research of over two decades has shown nitric oxide (NO) to be a ubiquitous modulator of biological phenomena from cell signal to effector and from physiology to pathophysiology. The involvement of NO in cardiovascular biology has contributed significantly to our understanding of complex disease states including atherosclerosis, systemic and pulmonary hypertension, endotoxic shock, preeclampsia, cardiomyopathy, myocardial infarction (MI), and cardiac allograft rejection. The dichotomy of effector function represents the “double-edged sword” of NO in biological systems. The balance between cytostatic and cytotoxic effects of NO may lie in the tissue concentration of NO produced, the particular NO synthase (NOS) isoform activation, and the complex interaction with other free radicals such as superoxide [1, 2]. All four NOS isoforms - endothelial NOS (eNOS), neuronal NOS (nNOS), inducible NOS (iNOS) and mitochondrial NOS (mtNOS) - have been shown to be present in the human myocardium and may be activated in response to hypoxia or ischemia. Studies of experimental myocardial infarction have shown an increased expression of iNOS, eNOS, and NO production in the heart, together with increased plasma concentrations of nitrate and nitrite, the oxidation products of NO. The isoform specific amount of NO generated may account, in part, for physiological versus pathological effects of NO; low concentrations are associated with cytostasis and high concentrations with cytotoxicity. A further explanation for the dichotomous effects of NO may lie in its complex interaction with reactive oxygen species (ROS), which is particularly pertinent in the context of ischemia–reperfusion. NO can interact in direct equimolar concentrations with superoxide to form peroxynitrite. The greater availability of superoxide may favor peroxynitrite production and toxicity. Thus, superoxide may be an important rate-limiting factor determining the protective versus toxic effects of NO. Although the interaction of NO with ROS is very complex, this simple relation may explain why despite the cytoprotective effects of NO against ischemia–reperfusion injury reported in the majority of animal studies, several authors reported cytotoxicity [3–7].

## Mechanisms of nitric oxide-mediated cardioprotection

The precise mechanisms whereby NO protects the myocardium against ischemia–reperfusion injury remain unclear. NO or its second messenger, cyclic guanosine monophosphate (cGMP), has been shown to exert a number of actions that would be expected to be beneficial against myocardial ischemia–reperfusion injury, including inhibition of Ca^2+^ influx into myocytes [8], antagonism of the effects of β-adrenergic stimulation [9], reduction in myocardial oxygen consumption [10, 11], and opening of sarcolemmal ATP-sensitive K^+^ (K^+^_ATP_) channel [12, 13]. NO protects the ischemic myocardium by stimulation of cyclooxygenase-2 (COX-2) activity with consequent production of cytoprotective prostanoids such as prostaglandin (PG) E2 and PGI [14]. This mechanism was identified by Shinmura et al. in the setting of late preconditioning, where inhibition of iNOS was found to abrogate prostanoid synthesis, whereas inhibition of COX-2 did not affect iNOS activity [14] but resulted in loss of protection, indicating that COX-2 activity is driven by iNOS-derived NO and is obligatorily required for iNOS to exert its cardioprotective effects [15].

Nitric oxide has also been suggested to protect against lethal ischemia–reperfusion injury by preventing the impairment of endothelium-dependent coronary vasodilation [16] and by reducing the “no reflow” phenomenon [17], the infiltration of leukocytes [18], the release of cytokines, and expression of adhesions molecules [19].

### NO and cardiomyocyte function

As mentioned above, NO via cGMP dose-dependently inhibits phosphodiesterase (PDE) and/or activates protein kinase G (PKG). At low NO/cGMP concentrations (in μM range), inhibition of PDEIII activity or direct activation of adenylyl cyclase [20] with subsequently increased cyclic adenosine monophosphate (cAMP) concentration and protein kinase A (PKA) activity increases cardiomyocyte function [22]. Additional mechanisms by which low NO/cGMP concentrations might increase cardiomyocyte function relate to a direct activation of ryanodine receptors or voltage-operated calcium channels [21]. At higher NO/cGMP concentrations (in μM range), activation of PKG inhibits voltage-dependent calcium channels [21, 22] and decreases myofilament calcium responsiveness by phosphorylation of troponin I [23]. While a higher NO/cGMP concentration also suppresses the increase in regional myocardial function during β-adrenergic stimulation [24], most likely by directly inhibiting ryanodine receptors, pharmacological blockade of endogenous NOS-dependent NO synthesis in pigs did not impact on adrenergic responsiveness [25]. Thus, only at high concentrations might NO/cGMP directly reduce cardiomyocyte function.

### NO and antioxidant activity

NO has been reported to be a free radical scavenger [26]. The antioxidant capacity of plasma was found to be doubled by the administration of NO donors. Moreover, these concentrations of the NO donors prevented reperfusion-induced mucosal injury, which has been shown to be mediated in large part by reactive oxygen metabolites [27]. A mechanism for superoxide anion scavenging by NO has not been clearly delineated. It is possible that production of NO in amounts exceeding local production of superoxide anion leads to accelerated decomposition of peroxynitrite to nitrate and nitrite, thus reducing tissue exposure to peroxynitrite and to the hydroxyl radical that can be formed from peroxynitrite. In addition to acting as a superoxide scavenger, NO may also have the ability to prevent superoxide production. Clancy et al. reported that NO could inhibit superoxide production from neutrophils by directly inhibiting nicotinamide adenine dinucleotide phosphate-oxidase (NADPH oxidase) [28].

### NO and anti-inflammatory effects

NO has for a long time been linked to the modulation of the immune response and effects on cell-mediated immunity may have a role in cytoprotection. High doses of NO have been shown to modulate the production of interleukin 12 negatively, thus reducing the T-helper cell 1 immune response [29]. In the context of inflammation, the endothelium plays a particularly important role in regulating the passage of blood and plasma constituents from the vasculature to the interstitium. The ability of these constituents to pass between adjacent endothelial cells is regulated by contractile elements within the endothelial cells. Contraction of these elements results in an increase in endothelial paracellular permeability. Thus, endothelial contraction contributes to edema formation in the context of inflammation. Various chemical mediators can increase endothelial permeability and promote edema formation, including histamine, leukotriene C4, and platelet-activating factor. The actions of NO on vascular permeability appear to be predominantly anti-inflammatory; that is, NO diminishes endothelial permeability. NO donors have been found to reduce edema formation in various experimental models, while inhibitors of NO synthesis can exacerbate edema formation [30, 31]. Infiltration of leukocytes to a site of injury or infection is a hallmark feature of inflammation, and one that can be profoundly influenced by NO. NO has been shown to inhibit the expression of the β-2 adhesion molecules on neutrophils [32]. Inhibition of NO synthesis results in a marked increase in leukocyte adherence to the endothelium [33], while adherence of leukocytes to the vascular endothelium in response to stimulation with a chemotactic factor can be markedly suppressed by NO donors [34, 35]. NO can also down-regulate neutrophil aggregation and secretion and may protect the neutrophil from damage induced by the potent reactive oxygen metabolites that it is capable of producing [36, 37]. NO can inhibit transcriptional events by inhibiting the transcription factor nuclear factor kappa B (NF-kB) [38]. NO appears to play an important role in regulation of adhesion molecules on the luminal surface of the endothelium. NO reduces P-selectin expression, while inhibitors of NO synthase elicit an increase in P-selectin expression and a corresponding increase in leukocyte adherence to the endothelium [33, 39]. As in the case of relaxant effects of NO on vascular smooth muscle, down-regulation of P-selectin expression by NO is mediated via soluble guanylate cyclase/ cGMP [40].

### NO and reduction in intracellular calcium overload

It is well known that accumulation of intracellular calcium (Ca^2+^) is lethal to cardiomyocytes [41, 42]. The consequence of excessive accumulation of intracellular Ca^2+^ during the early reperfusion phase leads to numerous secondary effects, including stimulation of contractile ‘‘rigor’’ [43, 44], mitochondrial dysfunction, over-stimulation of (Ca^2+^)-dependent enzymes, opening of the mitochondrial permeability transition pore [45], and induction of proapoptotic processes. NO reduces calcium influx into cardiomyocytes by modulating sarcolemmal Na^+^/H^+^ ion channel, mitochondrial Ca^2+^-ATP-ase, and by modulating ryanodine receptor type 2 (RyR2) Ca^2+^ release channels on the sarcoplasmatic reticulum. Therefore, NO reduces intracellular calcium overload and its complications.

### NO and apoptosis

In isolated cardiomyocytes and hearts, high NO concentrations (μM range) induce necrosis and apoptosis [46, 47]. The amount of necrosis and apoptosis critically depends on the energetic situation of the cardiomyocytes, with apoptosis favored at preserved ATP pools [48]. While the development of necrosis following NO application appears to be independent of cGMP, the development of apoptosis involves cGMP and subsequently activation of mitogen-activated protein kinases and transcription factors [46, 49, 50]. Most interestingly, the development of apoptosis following application of a NO donor is decreased, once cardiomyocytes or isolated hearts have been subjected to a preceding period of ischemia–reperfusion [46, 47], possibly by a diminished response of guanylyl cyclase to NO. NO can also directly inhibit apoptosis. Proposed mechanisms include the suppression of caspase 1 and 3 activity by NO-induced *S*-nitrosation; GMP-mediated suppression of calcium-mediated apoptotic cell death; and induction of the cytoprotective stress proteins heat shock protein 70 and heat shock protein 32 [51].

## Mitochondrial targets of cardioprotection and their interaction with NO

Mitochondria are the major site of cellular energy and production of adenosine triphosphate (ATP). The respiratory chain accepts electrons from nicotinamide dinucleotide (NADH)/H and flavine adenine dinucleotide (FADH)/H and transports them over 4 complexes ultimately onto oxygen, creating a proton gradient that then drives ATP production. Apart from ATP production and its role in cell function and survival, mitochondria are decisive elements for cell death by apoptosis, autophagy, and necrosis and, conversely, are targets for protection from cell death by NO-mediated mechanisms.

### Respiratory chain

The respiratory chain releases small amounts of ROS predominantly by complex I under physiological conditions [52]. Partial uncoupling of the respiratory chain protects against ischemia–reperfusion injury, supporting the importance of mitochondrial ROS for cardioprotection. During early reperfusion, ROS formation from various sources, including the respiratory chain, is largely augmented. At subcellular level, NO was shown to modulate mitochondrial function through reversible and irreversible interactions with respiratory chain complexes. Mitochondrial complexes I and III are major sources of pathological ROS production. Reversible inhibition of complex I has been proposed as a mechanism to achieve cardioprotection. The protective effects of complex I inhibition were described for different NO donors and observed during ischemic preconditioning in animal models [53–55]. Physiological concentrations of NO inhibit cytochrome oxidase (complex IV) in a reversible manner, in competition with oxygen. Reversible inhibition of complex I by *S*-nitrosation (NO-mediated modification of thiols) is cardioprotective by limiting excess ROS formation during reperfusion [56]. The reversible interaction may play an important part in the physiological regulation of mitochondrial function by reducing oxygen consumption without causing ATP depletion. This may be beneficial during ischemia [53, 57, 58]. Reversible suppression of mitochondrial respiration was shown to explain myocyte adaptation to chronic hypoxia without compromising cell survival or accelerating ATP depletion. Mitochondrial dysfunction is a critical component of ischemia–reperfusion injury, which is characterized by dissipation of the membrane potential, ATP depletion, induction of the transient mitochondrial permeability, and mitochondrial calcium overload. A number of data suggest that NO-induced depolarization of the mitochondrial membrane potential protects cardiomyocytes by reducing mitochondrial calcium overload during hypoxia–reoxygenation injury [54, 55, 59].

### Connexin 43

Connexin 43 is present at the inner mitochondrial membrane of cardiomyocytes. [60]. Reduction in connexin 43 abolishes the cardioprotection by ischemic preconditioning [61] but not postconditioning [62]. ROS formation and cardioprotection in response to diazoxide depend on mitochondrial connexin 43, suggesting that its function is to regulate the gating of mitochondrial K^+^_ATP_ channels [63]. Mitochondrial connexin 43 is targeted by several protein kinases, including glycogen synthase kinase 3 (GSK-3) [62].

### Mitochondrial potassium-ATP channel

The K^+^_ATP_ channel in the inner membrane is inhibited by ATP and activated by protein kinase C (PKC)-ε and PKG. The exact molecular composition of the K^+^_ATP_ channel and the participation of the sulfonylurea receptor subunit 2A (SUR2A) and the potassium channel proteins Kir6.1 and 6.2 remain elusive. A purified inner membrane fraction, including the adenine nucleotide transporter and succinate dehydrogenase, confers K^+^_ATP_ channel activity and is targeted by K^+^_ATP_ agonist/antagonist drugs [64]. Mitochondrial K^+^_ATP_ channels are causally involved in ischemic preconditioning and postconditioning [65, 66]. Sasaki et al. found that NO directly activates mitochondrial K^+^_ATP_ channels and potentiates the ability of diazoxide to open these channels [67]. Bell et al. demonstrated that nitric oxide can mediate cardioprotection in a dose-dependent fashion by an effect that may be related to mitochondrial membrane potential. Both cardioprotection and changes of mitochondrial membrane potential are sensitive to 5-hydroxy decanoate (5-HD), selective inhibitor of K^+^_ATP_ channel, and the cardioprotection appears independent of free radical synthesis [68]. Thus, on the basis of these results, it appears that the mitochondrial K^+^_ATP_ channel is a pivotal target for the protective effects of nitric oxide. Mitochondrial calcium overload is pathognomic of irreversible ischemia-reperfusion injury - the calcium paradox [69, 70]. Interestingly, mitochondrial K^+^_ATP_ channel openers attenuate the calcium paradox [71] and limit calcium accumulation in mitochondria by altering mitochondrial calcium homeostasis [72]. Given that opening of the mitochondrial permeability transition pore (PTP) is associated with triggering of apoptotic cell death cascades and that high calcium leads to opening of the PTP [73, 74], the finding that low-dose exogenous nitric oxide attenuates both the calcium paradox and the opening of the PTP is of great interest [75]. Since exogenous nitric oxide has been demonstrated to increase the open probability of mitochondrial K^+^_ATP_ channels [67], it is attractive to postulate that cardioprotective low-dose nitric oxide is initial mediated via the mitochondrial K^+^_ATP_ channel.

### Mitochondrial permeability transition pore

The mitochondrial PTP is a large-conductance megachannel putatively constituted by the voltage-dependent anion channel in the outer membrane, the adenine nucleotide transporter in the inner membrane, and cyclophilin D in the matrix [76]. Under physiological conditions, mitochondrial PTP is predominantly in a closed state. Although PTP opening is strongly inhibited by acidosis during ischemia, it is favored by ATP depletion, oxidative stress and high intramitochondrial Ca^2+^ concentrations, conditions all concurrent during myocardial reperfusion [77].

PTP opening is associated with mitochondrial swelling, outer membrane rupture, and the release of proapoptotic factors such as cytochrome c from the intermembrane space. Once released, cytochrome c activates caspase 9, which in turn activates caspase 3. This protease mediates the proteolytic cleavage of a range of proteins responsible for the rearrangement of the cytoskeleton, plasma membrane, and nucleus that are characteristic of apoptosis [78, 79]. Opening of the mitochondrial PTP is considered a key event in cell death after ischemia–reperfusion [74, 80]. Inhibition of PTP opening is cardioprotective, but transient opening of PTP is required for cardioprotection [81]. Mitochondrial PTP opening depends on NO. Low levels of NO prevent PTP opening, whereas high NO levels accelerate PTP opening and cytochrome c release [56]. Part of the apparent paradox may be methodological in nature. Subsarcolemmal and interfibrillar mitochondria differ in their morphology and function. It is possible that sarcolemmal mitochondria serve a signaling function, whereas interfibrillar mitochondria are targets of damage and protection from it.

These were the many positive effects of NO in ischemia–reperfusion (IR) injury. But on the other hand, NOS enzymes have also been shown to play a deleterious role in IR injury. For example, iNOS-deficient mice were shown to have lower mortality and enhanced left ventricular contractility when compared with wild-type mice after coronary occlusion [82]. Also, exposing mitochondria to high concentrations of NO (μM) has been shown to initiate mitochondrial PTP opening [75]. These results, along with other studies, have defined NO as a dual-faced molecule in IR injury, which contributes to both cardioprotective and deleterious signaling pathways within the myocardium. In this regard, understanding how to deliver NO (i.e., timing, concentration, location) may facilitate beneficial therapeutic exploitation of NO signaling in IR injury, while minimizing the deleterious effects of NO. With respect to these experimental results, it seems to indicate that the protective effects of low-dose nitric oxide in the whole heart are not mediated by their generation, which appears contrary to a previous report using exogenous nitric oxide used as a trigger of preconditioning [83] and also postconditioning. The discrepancy would suggest that exogenous nitric oxide as a cardioprotective agent mediates protection via different mechanisms to those recruited by transient exposure of the heart to nitric oxide to trigger preconditioning; preconditioning requires up-stream signaling to result in a cardioprotective “memory.” In the paradigm presented in this report, nitric oxide is free to act directly upon end-effector targets downstream of preconditioning- and postconditioning recruited pathways, without the need to recruit preconditioning memory, and therefore no need for free radicals to achieve this state.

In 2011, Minamishima et al. found that NO inhalation at 40 ppm for 23 h starting 1 h after successful cardiopulmonary resuscitation markedly improved myocardial and neurological function and survival rate 10 days after cardiac arrest in mice [57]. Based on these experimental and clinical results, we formulated hypothesis that NO donors may be very beneficial in acute heart failure with cardiogenic shock and cardiopulmonary resuscitation treatment.

## Theoretical basis for the value of NO donation in ischemia–reperfusion injury

According to current recommendations, intravenous nitroglycerine is contraindicated when systolic blood pressure (BP) is below 90 mmHg. Hemodynamic properties of vasodilators, and NO donors in particular, were extensively studied in the 1970s and 1980s, although usually not in terminal patients with no BP. Franciosa et al. [84] reported that intravenous sodium nitroprusside increased cardiac output and decreased wedge pressure in 15 patients with acute MI and elevated left ventricular filling pressure. Their BP was not allowed to fall below 95 mmHg, with the average drop in systolic BP at only 26 mmHg. Similar results were achieved in severe heart failure secondary to ischemic or dilated cardiomyopathy. In 1984, Bayley et al. [85] evaluated incremental doses of intravenous nitroglycerine in patients with left ventricular failure. The maximal hemodynamic benefit, in terms of decrease in wedge pressure and increase in cardiac index, was obtained at 160 μg/min, which represented the highest dose tested. Cotter et al. [86] randomized patients with pulmonary edema into cohorts receiving isosorbide dinitrate at 3-mg bolus administered intravenously every 5 min versus traditional treatment using low doses of isosorbide, furosemide, and morphine. BP was not allowed to drop below 90 mmHg. Mechanical ventilation was required in 13 % of the high-dose nitrate group and in 40 % of the traditional group. MI occurred in 17 and 37 %, respectively [86]. The poor prognosis of cardiac arrest is driven primarily by brain and heart injury. Except for hypothermia, no beneficial postresuscitation therapies have emerged since the description of cardiopulmonary resuscitation (CPR) 50 years ago. The present postresuscitation care is largely supportive. The role of NO donors would be of great importance in this setting. In man, myocardial dysfunction is common in cardiac arrest and strongly associated with mortality, yet it may be made ultimately reversible. The molecular mechanisms of myocardial stunning after cardiac arrest remain unknown, but loss of excitation–contraction coupling is believed to result from ROS injury and calcium-mediated proteolysis. Based on the above findings, we formulated the hypothesis of the complex action of NO donors in cardiac arrest and acute heart failure and cardiogenic shock treatment. On the one hand, NO donors may increase cardiac output produced by rapid vasodilatation in a heart operating at the extreme of the Frank-Starling curve. In heart failure with or without acute MI, vasodilators have over a long time been shown to decrease left ventricular filling pressure and systemic vascular resistance while increasing the cardiac index. The more severe the failure, the more beneficial the effect of vasodilators. On the other hand, NO donors by inhibiting mitochondrial complex I and reducing ROS production may mitigate cardiac stunning and improve systolic function, cardiac output, and ultimately prove lifesaving. NO also stabilizes the mitochondrial membrane and directly or indirectly inhibits proapoptotic processes. It is tempting to consider the possibility that NO regulates also calcium levels in the mitochondria by modulating the activity of the mitochondrial calcium uniporter, a mechanism protecting against lethal mitochondrial calcium overload. Furthermore, exogenous administration of NO donor could lead to reversible inhibition of mtNOS in particular, and thus to a reduction in peroxynitrite formation. These mechanisms act together in synergy and are complementary. Vasodilatation of the coronary artery ensures adequate myocardial perfusion. Furthermore, reversible inhibition of mitochondrial complex I blocks production of ROS in reperfusion. We assume that the synergy of these mechanisms is able to improve systolic function and consequently increase systolic BP. This can be explained by the so-called paradoxical increase in systolic blood pressure after bolus quantity of NO donors. Of course, according actually facts NO donors in the peripheral parts of the circulation cause also vasodilation. On the other side, we assume that NO increases vascular sensitivity to noradrenaline and sympathetic stimulation. In this way, we explain the increase and stabilization of diastolic BP after bolus administration of NO donors in cardiopulmonary resuscitation.

## Conclusions

In conclusion, the action of NO donors in cardiac arrest may be summarized in three points: (1) a direct hemodynamic effect mediated through vasodilation of coronary arteries in cooperation with, (2) direct effect on improving cardiac function and cardiac output through above mentioned molecular mechanisms, and (3) at the same time, an increase in vascular sensitivity to sympathetic stimulation could lead to increase in diastolic blood pressure. This hypothesis must of course be verified by several clinical studies. If confirmed, it may mean a major breakthrough in the treatment for cardiac arrest and cardiopulmonary resuscitation that would result not only in better effects on cardiac function but prove even lifesaving.
